# Synergy in Dual Engagement of Extrinsic and Intrinsic Apoptosis Pathways by Bleomycin and Panobinostat in Hepatocellular Carcinoma and Targeting Mcl-1-Dependent Apoptosis Resistance

**DOI:** 10.3390/biomedicines14081805

**Published:** 2026-08-11

**Authors:** Patricia Mester, Lena Aschenbrenner, Vlad Pavel, Philipp Heumann, Elisabeth Aschenbrenner, Kirstin Pollinger, Karsten Gülow, Claudia Kunst, Tobias Schilling, Martina Müller

**Affiliations:** 1 Department of Internal Medicine I, Gastroenterology, Hepatology, Endocrinology, Rheumatology, Immunology and Infectious Diseases, University Hospital Regensburg, Franz-Josef-Strauß-Allee 11, 93053 Regensburg, Germany; vlad.pavel@ukr.de (V.P.); philipp.heumann@ukr.de (P.H.); elisabeth.aschenbrenner@ukr.de (E.A.); kirstin.pollinger@ukr.de (K.P.); karsten.guelow@ukr.de (K.G.); claudia.kunst@ukr.de (C.K.); martina.mueller-schilling@ukr.de (M.M.); 2 Department of Internal Medicine III, Hematology and Oncology, University Hospital Regensburg, 93053 Regensburg, Germany; lena.aschenbrenner@ukr.de; 3 Department of Emergency and Intensive Care Medicine, Klinikum Stuttgart, 70174 Stuttgart, Germany; t.schilling@klinikum-stuttgart.de

**Keywords:** hepatocellular carcinoma (HCC), apoptosis, panobinostat, bleomycin, Bcl-2 family, Bcl-XL, Mcl-1

## Abstract

**Background**: Hepatocellular carcinoma (HCC) remains a major clinical challenge due to its pronounced molecular heterogeneity and frequent resistance to conventional therapies. A key driver of therapeutic failure is the overexpression of the anti-apoptotic proteins myeloid cell leukemia-1 (Mcl-1) and B-cell lymphoma-extra large (Bcl-XL), which collectively maintain mitochondrial integrity and promote tumor cell survival. **Methods**: In this study, we evaluated a rational combination strategy targeting these complementary survival pathways using the histone deacetylase inhibitor panobinostat and the DNA-damaging agent bleomycin in HepG2 cells, a p53-functional HCC cell model. **Results**: In HepG2 cells, each agent alone produced only limited cytotoxicity, whereas their combination resulted in a marked and synergistic induction of apoptosis. This was shown by increased Annexin V positivity, mitochondrial outer membrane permeabilization (MOMP), and activation of caspases-8, -9, and -3 as well as cleavage of poly(ADP-ribose) polymerase (PARP). Mechanistically, panobinostat reduced Bcl-XL expression and primed mitochondria for apoptosis but simultaneously triggered compensatory upregulation of Mcl-1, representing an adaptive resistance response within this experimental system. Bleomycin effectively counteracted this escape mechanism by suppressing Mcl-1 induction, thereby lowering the apoptotic threshold and enabling mitochondrial permeabilization. In parallel, combined treatment potentiated caspase-8 cleavage, suggesting an additional caspase-8-associated apoptotic signal that amplified caspase-3/PARP execution. Pharmacological inhibition with zVAD-FMK confirmed that the observed cell death was predominantly caspase-dependent, supporting a coordinated engagement of both intrinsic and extrinsic apoptotic pathways. In summary, the combination of panobinostat and bleomycin overcomes anti-apoptotic defenses in HepG2 cells through synergistic and coordinated disruption of mitochondrial survival checkpoints and dual apoptosis pathway activation. **Conclusions**: By blocking a compensatory Mcl-1 escape response while simultaneously engaging extrinsic apoptosis signaling, this strategy produces potent synergistic cell death in this defined p53-functional HCC model and represents a promising mechanistic proof of concept that warrants further validation in additional molecularly diverse HCC models before broader translational conclusions can be drawn.

## 1. Introduction

Hepatocellular carcinoma (HCC) is the most common primary liver malignancy and remains a leading cause of cancer-related mortality worldwide [1,2]. Despite advances in prevention and therapy, its global burden continues to rise [1]. Historically driven by chronic hepatitis B and C virus infection, the etiological landscape of HCC is undergoing a major shift due to the rapidly growing prevalence of metabolic dysfunction-associated steatotic liver disease (MASLD), which has emerged as a dominant risk factor in Western countries and is increasingly recognized worldwide [3]. Regardless of etiology, the overall 5-year survival rate of HCC remains poor, at approximately 18% [4,5,6,7,8].

HCC development is driven by a complex interplay of genetic and epigenetic alterations that promote malignant transformation, tumor progression, and therapy resistance [9]. In clinical practice, treatment allocation is guided by the Barcelona Clinic Liver Cancer (BCLC) staging system, which integrates tumor burden, liver function, and patient performance status [9,10,11,12]. Curative-intent approaches, including surgical resection, ablation, and liver transplantation, are reserved for early-stage disease (BCLC 0/A). Patients with intermediate-stage disease (BCLC B) who are unsuitable for locoregional therapies and those with advanced-stage disease (BCLC C) are candidates for TACE (transarterial chemoembolization) and systemic therapy [11,13]. The updated BCLC classification for HCC introduces the concepts of downstaging and treatment stage migration to allow more flexible, patient-centered management. Downstaging means that patients with more advanced tumors can receive therapy (e.g., locoregional treatments) to reduce tumor burden. If successful, they may be reclassified into an earlier BCLC stage and become eligible for curative options such as resection, ablation, or liver transplantation. Treatment stage migration allows progression beyond the standard treatment recommended for a given BCLC stage. If the first-line therapy for that stage is ineffective, contraindicated, or not tolerated, alternative treatments from other stages can be applied. Overall, these concepts emphasize dynamic staging, individualized treatment decisions, and adaptation of therapy over time rather than rigid adherence to a single stage-treatment algorithm [10].

In accordance with current EASL recommendations, first-line systemic treatment consists of immune checkpoint inhibitor-based combinations, including atezolizumab plus bevacizumab and tremelimumab plus durvalumab or ipilimumab plus nivolumab [11,14,15,16,17]. Tyrosine kinase inhibitors remain an established alternative for patients who are ineligible for immunotherapy [11,14,18,19,20]. Nevertheless, long-term disease control remains limited, highlighting the need for new therapeutic strategies that can overcome intrinsic and acquired resistance mechanisms in HCC [11,14]. Approaches currently focus on combination therapy with TACE and systemic treatment. In the LEAP-012 trial (TACE plus pembrolizumab plus lenvatinib vs. TACE alone) and the EMERALD-1 trial (TACE plus durvalumab plus bevacizumab vs. TACE alone), a statistically significant prolongation of progression-free survival (PFS) was demonstrated for the multimodal treatment concept [15,21]. Importantly, no statistically significant improvement in overall survival has been shown so far. Further clinical trials and the development of additional multimodal therapeutic strategies are therefore essential.

Bleomycin is currently not included in standard systemic treatment regimens for HCC. However, emerging clinical evidence suggests a potential role for bleomycin in locoregional therapy. In patients, refractory to conventional doxorubicin-based (TACE), bleomycin-based TACE has demonstrated superior objective response rates, progression-free survival, and overall survival, with a comparable safety profile [22]. These findings indicate that bleomycin may represent a viable alternative in selected HCC populations.

The principal dose-limiting toxicity of bleomycin is pulmonary injury, which may manifest as pneumonitis or fibrosis in a dose- and age-dependent manner and can be fatal in rare cases [23,24]. Accordingly, careful monitoring of pulmonary function is required during treatment. At the molecular level, bleomycin exerts its cytotoxic effects through the induction of DNA damage via free radical formation, resulting in single- and double-strand DNA breaks. This activates the DNA damage response, prominently involving p53 signaling, leading to transcriptional upregulation of pro-apoptotic mediators such as CD95 (Fas) [25,26]. Consequently, bleomycin can trigger apoptosis through both extrinsic CD95-mediated signaling and the intrinsic mitochondrial pathway [25,27].

Histone deacetylases (HDACs) regulate chromatin accessibility and gene transcription through deacetylation of histone proteins [28]. Although histone deacetylase inhibitors (HDACis) are not part of current guideline-recommended HCC therapy, they are under active investigation as potential anticancer agents. Preclinical studies have demonstrated that HDACis suppress HCC cell proliferation, induce apoptosis, and enhance the activity of established therapies, including tyrosine kinase inhibitors and immune checkpoint inhibitors [29,30,31,32,33,34]. Selective HDAC8 inhibition has been shown to augment antitumor immunity and improve responses to checkpoint blockade in experimental HCC models [34]. Early-phase clinical trials evaluating HDACis such as belinostat and resminostat have reported manageable toxicity profiles and antitumor activity, especially in combination regimens [30,32].

Histone deacetylase inhibitors (HDACis) modulate chromatin structure and transcription and thereby influence key oncogenic pathways, including proliferation, cell cycle regulation, and apoptosis [35]. HDACis can engage both intrinsic and extrinsic apoptotic signaling and have been shown to sensitize tumor cells to DNA-damaging agents [36]. In particular, chromatin relaxation induced by HDAC inhibition enhances accessibility of DNA-targeting compounds and has been associated with synergistic antitumor activity in preclinical models [36,37]. Panobinostat (LBH589), a potent pan-HDAC inhibitor approved for multiple myeloma, exhibits strong antitumor activity in HCC models by inducing apoptosis and autophagy, modulating non-coding RNA networks, and inhibiting angiogenic signaling pathways [36,37,38].

Epigenetic plasticity is a hallmark of HCC and contributes to the acquisition of apoptosis resistance, thereby promoting tumor progression and limiting therapeutic efficacy [39]. Accordingly, the identification of molecular vulnerabilities and predictive biomarkers that enable rational combination strategies remains a major clinical priority [40]. Among regulated cell death pathways, apoptosis represents a central tumor-suppressive mechanism whose impairment is strongly associated with treatment failure in HCC [41].

Intrinsic (mitochondrial) apoptosis is frequently dysregulated in cancer and is tightly controlled by the BCL-2 protein family [42]. Anti-apoptotic members, particularly Mcl-1 and Bcl-XL, are constitutively expressed in hepatocytes and are critical for maintaining hepatic homeostasis [42,43,44]. However, their persistent expression in malignant hepatocytes contributes to apoptosis resistance [42,45].

Based on these considerations, we hypothesized that combining panobinostat with the DNA-damaging agent bleomycin would lower the apoptotic threshold in a defined p53-functional HCC cell model by simultaneously disrupting epigenetic survival programs and activating mitochondrial and caspase-8-associated apoptotic signaling, thereby overcoming intrinsic resistance mechanisms within this specific experimental context. Specifically, we postulated that panobinostat-induced chromatin remodeling would sensitize HepG2 cells to bleomycin-mediated DNA damage, while bleomycin would counteract compensatory anti-apoptotic adaptations, resulting in amplified caspase-dependent apoptotic execution. To test this hypothesis, we evaluated the individual and combined effects of panobinostat and bleomycin on cell viability, mitochondrial integrity, caspase activation, and key regulators of apoptotic signaling in HepG2 cells.

## 2. Materials and Methods

### 2.1. Cell Culture

Human HepG2 cells (American Type Culture Collection (ATCC) HB-8065, Manassas, VA, USA) were cultured in RPMI-1640 medium (Sigma-Aldrich, Biochemie GmbH, Hamburg, Germany) supplemented with 10% fetal bovine serum (Thermo Fisher Scientific GENEART GmbH, Regensburg, Germany) without antibiotics at 37 °C in humidified atmosphere of 5% CO_2_. Human Hep3B cells (American Type Culture Collection (ATCC) HB-8064) were cultured in MEM medium (Sigma-Aldrich, Biochemie GmbH, Hamburg, Germany) supplemented with 10% fetal bovine serum (Thermo Fisher Scientific GENEART GmbH, Regensburg, Germany) without antibiotics at 37 °C in humidified atmosphere of 5% CO_2_. Human Huh7 (Cytion, Heidelberg, Germany) and human Huh6 cells (Cytion, Heidelberg, Germany) were cultured in DMEM high glucose medium (Sigma-Aldrich, Biochemie GmbH, Hamburg, Germany) supplemented with 10% fetal bovine serum (Thermo Fisher Scientific GENEART GmbH, Regensburg, Germany) without antibiotics at 37 °C in humidified atmosphere of 5% CO_2_.

### 2.2. Treatment with Anticancer Drugs

HepG2 cells were exposed to bleomycin (TargetMol, Wellesley Hills, MA, USA) at 0.0028–0.014 mg/mL (2–10 µM) and/or panobinostat (Selleckchem; Absource Diagnostics GmbH, Munich, Germany) at 350–1750 ng/mL (1–5 µM). Notably, the bleomycin concentrations used in vitro were deliberately kept far below reported therapeutically relevant serum levels (1.5–3.0 mg/mL; ~10^2^–10^3^-fold lower) [17,43].

Panobinostat serum concentrations in patients have been reported in the low-ng/mL range (8–20 ng/mL) [44,45]. Overall, these intentionally modest, submaximal exposure conditions were selected to limit dominant single-agent effects and thereby sensitively assess potential combinatorial (synergistic) activity between two clinically approved agents, in line with a repurposing-oriented strategy aimed at identifying efficacy at reduced dosing with the longer-term goal of mitigating dose-limiting toxicities.

### 2.3. Cell Death Analysis

#### 2.3.1. Assessment of Apoptotic Signaling and Cell Death

Activation of intrinsic apoptotic signaling was assessed by immunoblot analysis of BCL-2 family proteins (BCL-XL and MCL-1) and by detection of caspase-9 cleavage. Activation of extrinsic apoptotic signaling was evaluated by Western blot analysis of caspase-8 cleavage. Execution of apoptosis was determined by immunoblot detection of caspase-3 and poly(ADP-ribose) polymerase (PARP) cleavage.

#### 2.3.2. Measurement of Mitochondrial Membrane Potential (Δψm)

Mitochondrial membrane potential (Δψm) was quantified by flow cytometry using the cell-permeant fluorescent dye tetramethylrhodamine ethyl ester (TMRE; Invitrogen, Waltham, MA, USA; T669). TMRE selectively accumulates in polarized mitochondria, and reduced dye uptake was interpreted as mitochondrial depolarization.

A 50 mM TMRE stock solution was prepared in anhydrous DMSO, aliquoted, stored at −20 °C, protected from light, and thawed immediately before use. Cells were pretreated with the indicated drugs for 24 h, followed by incubation with TMRE (30 µM) for 30 min at 37 °C in 5% CO_2_. Vehicle controls received matched DMSO (≤0.1% *v*/*v*).

After staining, cells were washed once with PBS, resuspended in ice-cold PBS supplemented with 10% fetal calf serum (FCS), and analyzed on an LSRFortessa™ Cell Analyzer (BD Biosciences, Heidelberg, Germany). Debris and doublets were excluded by FSC/SSC gating and singlet discrimination. TMRE fluorescence was recorded using the 561 nm laser and is reported as median fluorescence intensity (MFI), normalized to the respective vehicle control.

#### 2.3.3. Flow Cytometric Analysis of Cell Death

Cell death was quantified by Annexin V/propidium iodide exclusion staining. Cells were harvested 12 h and 24 h after treatment with bleomycin, panobinostat, or the combination of both; stained with Annexin V–FITC and DAPI (Sigma-Aldrich, Hamburg, Germany) according to the manufacturer’s instructions; and analyzed by flow cytometry using an LSRFortessa™ Cell Analyzer. Viable cells were defined as Annexin V^−^/DAPI^−^.

To allow comparison across independent experiments, specific cell death was calculated using the following formula [46]:

$$\mathrm{Specific}\;\mathrm{cell}\;\mathrm{death}\;(\%)=\frac{\mathrm{experimental}\;\mathrm{cell}\;\mathrm{death}\;(\%)\;-\;\mathrm{spontaneous}\;\mathrm{cell}\;\mathrm{death}\;(\%)}{100\;-\;\mathrm{spontaneous}\;\mathrm{cell}\;\mathrm{death}\;(\%)}\times 100$$

#### 2.3.4. Caspase Inhibition zVAD-FMK

For pharmacological inhibition of caspase activity, the cell-permeable irreversible pan-caspase inhibitor zVAD-FMK (Z-Val-Ala-Asp(OMe)-fluoromethyl ketone; Selleck Chemicals GmbH; S7023) was used. A 50 mM stock solution was prepared in DMSO, aliquoted, and stored at −20 °C. Cells were pretreated with zVAD-FMK (50 µM) for 20 min prior to stimulation with bleomycin and panobinostat and maintained in the presence of the inhibitor throughout the experiment (12 h and 24 h). Vehicle controls received matched DMSO at the same final concentration.

### 2.4. Western Blot

For protein isolation, cells were washed with PBS and lysed in RIPA buffer containing protease and phosphatase inhibitors (cOmplete, PhosSTOP; Roche Diagnostics GmbH, Mannheim, Germany). Lysates were incubated on ice for 1 h and centrifuged at 14,000 rpm for 1 h at 4 °C. Protein-containing supernatants were collected, separated by SDS–PAGE, and transferred to PVDF membranes. Each gel was loaded with 15–30 µL of the sample.

For protein extraction, cell culture medium was removed, and cells were collected by centrifugation for 5 min at 1300 rpm. Cell pellets were resuspended in 900 µL PBS, transferred to microcentrifuge tubes, and centrifuged for 5 min at 1800 rpm. After removal of the supernatant, pellets were lysed in RIPA buffer for 1 h. Lysates were subsequently centrifuged for 1 h at 14,000 rpm and 4 °C, and the supernatants were transferred to fresh tubes for immediate analysis or storage at −20 °C.

Protein concentrations were determined using the Pierce™ Rapid Gold BCA Protein Assay Kit (Thermo Fisher Scientific). RIPA lysates were diluted 1:3 and analyzed in duplicate in 96-well plates. Bovine serum albumin (BSA; 200–2000 µg/mL) was used to generate a standard curve. Subsequently, 200 µL of a 1:50 mixture of reagents A and B were added to each well, followed by incubation for 5 min at 37 °C. Absorbance was measured at 492 nm using EMAX Plus software (version 6.5.1).

For immunoblot analysis, 10–25 µg of protein was adjusted to a final volume of 15–30 µL in RIPA buffer, supplemented with 4 µL 6× Laemmli sample buffer containing dithiothreitol, and denatured at 95 °C.

Antibodies against full-length caspase-9 (9508, Cell Signaling Technology, Danvers, MA, USA) and cleaved caspase-9 (7237, Cell Signaling Technology, Danvers, MA, USA), full-length and cleaved caspase-3 (9662, Cell Signaling Technology, Danvers, MA, USA), full-length and cleaved caspase-8 (9746, Cell Signaling Technology, Danvers, MA, USA), full-length and cleaved PARP (9542, Cell Signaling Technology, Danvers, MA, USA), Mcl-1 (4572, Cell Signaling Technology, Danvers, MA, USA), and Bcl-XL (2762S, Cell Signaling Technology, Danvers, MA, USA) were used at 1:1000 dilution. Secondary antibodies against rabbit or mouse IgG (Sigma-Aldrich, St. Louis, MO, USA) were used at 1:10,000 and 1:5000 dilutions, respectively. Protein band intensities were quantified using Image Lab Software (Version 6.0.1. build 34, Bio-Rad Laboratories Inc., Munich, Germany). PageRuler™ Plus Prestained Protein Ladder (Thermo Fisher Scientific, Regensburg, Germany) was used as a molecular weight marker.

### 2.5. Transfection

#### 2.5.1. Transient Overexpression of Mcl-1 in HepG2 Cells

HepG2 cells were seeded at a density of 1 × 10^6^ cells per 6 cm culture dish in 4 mL RPMI medium. After 24 h, cells were transiently transfected with either a human MCL1 cDNA ORF expression plasmid carrying a C-terminal FLAG tag (HG10240-CF; Sino Biological, Beijing, China) or the corresponding empty-vector control (pCMV3-FLAG).

For transfection, 2 µg plasmid DNA was diluted in 500 µL Opti-MEM™ medium (Thermo Fisher Scientific), followed by the addition of 15 µL X-tremeGENE™ Transfection Reagent (Roche Diagnostics, Mannheim, Germany). After gentle mixing, complexes were allowed to form for 20 min at room temperature and were added dropwise to the cells. Twenty-four hours after transfection, cells were treated with bleomycin and panobinostat as indicated. Following a further 24 h of treatment, cells were harvested, and protein lysates were prepared for subsequent analyses.

#### 2.5.2. Transient Silencing of Mcl-1 in HepG2 Cells

HepG2 cells were seeded at a density of 1 × 10^6^ cells per 6 cm culture dish in 4 mL RPMI medium. After 24 h, cells were transiently transfected with either a Silencer™ Validated siRNA targeting human MCL1 (AM51334; Invitrogen, Waltham, MA, USA) or Silencer™ Select Negative Control No. 2 siRNA (4390847; Invitrogen).

SiRNAs were reconstituted in nuclease-free water to a stock concentration of 100 µM. For each 6 cm dish, 2 µL siRNA stock solution was diluted in 300 µL Opti-MEM™ medium. In parallel, 30 µL TransFectin™ Transfection Reagent (Bio-Rad, Hercules, CA, USA) was diluted in 300 µL Opti-MEM™ medium. The diluted TransFectin reagent was added to the siRNA solution, gently mixed, and incubated for 20 min at room temperature to allow complex formation. The resulting complexes were added dropwise to the cells.

Twenty-four hours after transfection, cells were treated with bleomycin and panobinostat as indicated. After a further 24 h, cells were harvested, and protein lysates were prepared for subsequent analyses.

### 2.6. Statistical Analysis

Data are presented as mean ± SD. Normality was tested with the Shapiro–Wilk test. Between-group comparisons used Welch’s *t* test and one-way ANOVA with Dunnett’s two-sided post hoc tests (all computed in SigmaPlot v15; α = 0.05). Representative flow cytometry plots were selected by median fluorescence intensity within groups. Synergy analyses were performed in SAS 9.4 and modeled outcomes as continuous variables on the original scale using two-way factorial linear mixed models (REML) with fixed effects for bleomycin, panobinostat, and their interaction; non-additivity was assessed via the type III test of the interaction. For selected dose pairs, interaction magnitude and direction were quantified with pre-specified difference-in-differences contrasts, reported with two-sided *p* values and 95% CIs.

In this framework, the interaction term tests whether the observed combined treatment effect deviates from simple additivity of the two single-agent effects on the original apoptosis percentage-point scale. Accordingly, in the context of this analysis, the term “synergy” refers to statistically significant supra-additivity/non-additivity within the tested dose range and under the defined experimental conditions. This factorial interaction approach is conceptually distinct from classical pharmacological reference models such as Bliss independence, Loewe additivity, and Highest Single Agent (HSA), which rely on different assumptions and are typically most informative in the context of more extensive dose–response matrices. In particular, formal Loewe additivity or combination-index analysis requires sufficient single-agent dose–response information to define dose equivalence across both compounds. Because the present study was designed as a mechanistically focused analysis of selected submaximal dose combinations rather than as a comprehensive drug-combination matrix, classical Bliss-, Loewe-, and HSA-based synergy scores were not calculated.

## 3. Results

### 3.1. Bleomycin and Panobinostat Synergistically Induce Apoptosis in HepG2 Cells

**Figure 1:**
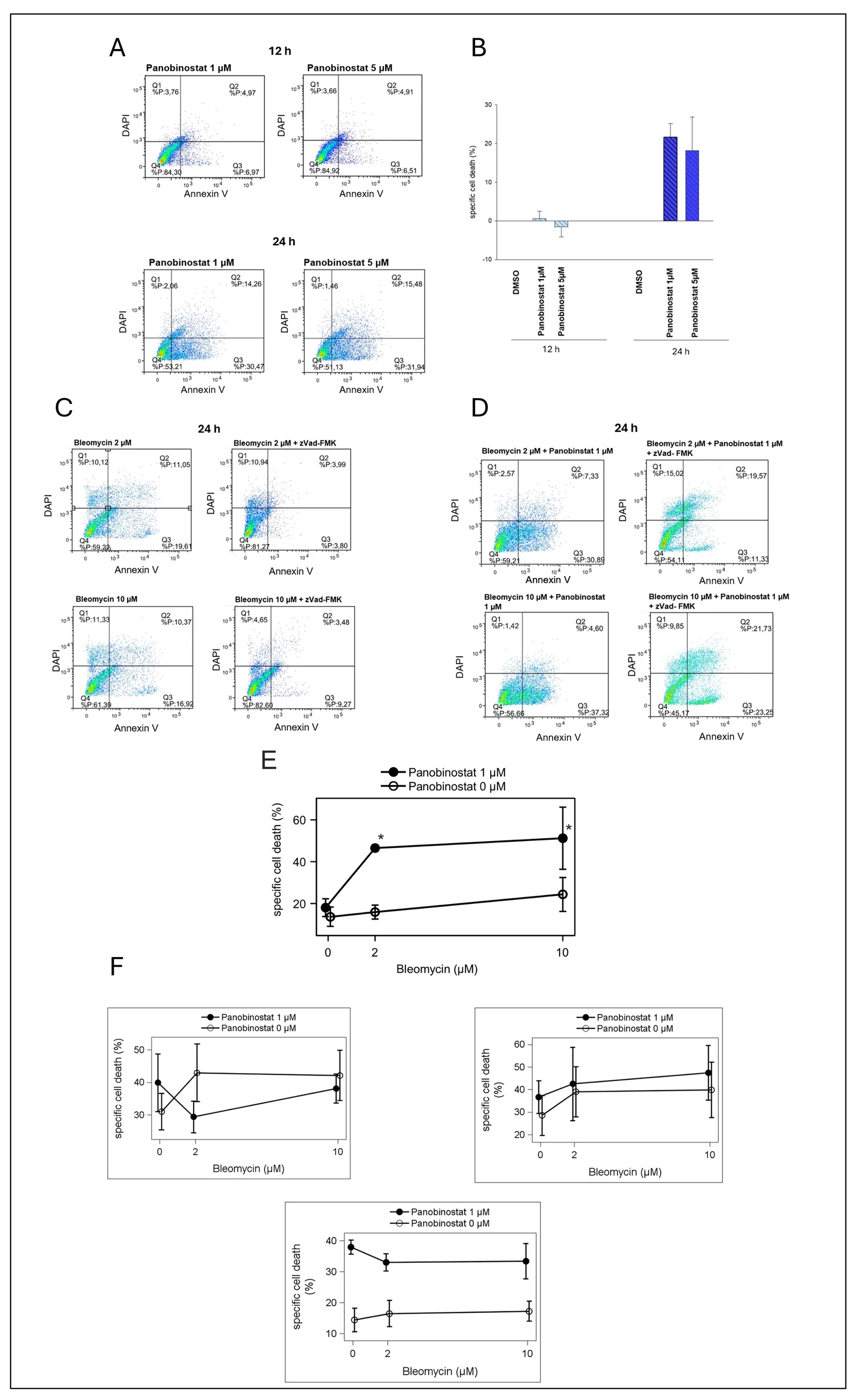
Panobinostat and its combination with bleomycin synergistically induces apoptosis in HepG2 cells but alone or in combination with bleomycin does not induce apoptosis in Hep3B, Huh6, or Huh7 cells. (**A**,**C**,**D**) Cell death assay for HepG2 cells incubated with bleomycin (2 µM/10 µM), panobinostat (1 µM/5 µM) or a combination of both for 12–24 h. DMSO was used as a vehicle control. Cells were analyzed using flow cytometry after Annexin V/DAPI staining. (**B**,**E**,**F**) Quantification of specific cell death and synergy calculation determined from the Annexin V/DAPI assay in panels A, C and D (*n* = 3, mean ± SD, Welch’s *t*-test; *: *p* = <0.05, **: *p* = <0.01 and ***: *p* = <0.001).

Since panobinostat has been reported to induce apoptosis in hepatocellular carcinoma (HCC) cell lines [38,47,48], we first sought to verify this effect in our HepG2 cell model. HepG2 cells were treated with 1 µM or 5 µM panobinostat, and cell death was analyzed by flow cytometry after 12 h and 24 h. The majority of treated cells were Annexin V-positive, indicating apoptotic cell death. Notably, panobinostat-induced apoptosis became evident predominantly after 24 h, with 1 µM eliciting a slightly stronger apoptotic response than 5 µM (Figure 1A,B).

To assess the apoptotic effects of bleomycin, HepG2 cells were exposed to 2 µM or 10 µM bleomycin for 24 h. Flow cytometric analysis revealed Annexin V-positive cells as the predominant population at both concentrations, confirming apoptosis as the principal mode of cell death (Figure 1C). Approximately 11% of cells were double-positive for Annexin V and DAPI, consistent with late-stage apoptosis or secondary necrosis.

To determine caspase dependency, cells were co-treated with the pan-caspase inhibitor zVAD-FMK. Caspase inhibition markedly attenuated bleomycin-induced cell death (Figure 1C), indicating a caspase-dependent mechanism. Notably, when bleomycin was combined with 1 µM panobinostat, total cell death increased from approximately 10% to approximately 40%, demonstrating a significantly enhanced apoptotic response compared with either single treatment alone.

### 3.2. Statistical Assessment of Bleomycin–Panobinostat Synergy in Apoptosis Induction in HepG2 Cells

All six treatment combinations (bleomycin 0/2/10 µM × panobinostat 0/1 µM) were represented with *n* = 4 measurements each and no missing apoptosis values. In the factorial model, apoptosis was significantly affected by bleomycin (F(2,18) = 17.93, *p* < 0.0001) and by panobinostat (F(1,18) = 45.41, *p* < 0.0001). Importantly, the bleomycin × panobinostat interaction was significant (F(2,18) = 7.18, *p* = 0.0051), demonstrating a non-synergistic interaction between the two treatments on the percentage-point scale.

Consistent with this interaction, the estimated effect of panobinostat on apoptosis depended on the bleomycin concentration. In the absence of bleomycin, panobinostat produced only a small, non-significant increase in apoptosis (+4.37 percentage points; *p* = 0.4208). In contrast, panobinostat markedly increased apoptosis when combined with bleomycin, yielding increases of +30.71 percentage points at bleomycin 2 µM (*p* < 0.0001) and +26.88 percentage points at bleomycin 10 µM (*p* < 0.0001). Pre-specified DiD contrasts confirmed supra-additivity: the synergy estimate was 26.33 percentage points at bleomycin 2 µM (95% CI 10.56–42.10; *p* = 0.0025) and 22.50 percentage points at bleomycin 10 µM (95% CI 6.73–38.27; *p* = 0.0077). Together, these results show that panobinostat enhances bleomycin-induced apoptosis in a synergistic manner at the tested concentrations.

To assess whether the response to combined bleomycin and panobinostat treatment depends on the p53 pathway, we repeated the experiments under identical conditions in three additional liver cancer cell lines with distinct p53-family backgrounds. Neither bleomycin nor panobinostat alone exerted a significant main effect. However, a significant negative interaction between both treatments was detected (type III test, *p* = 0.0164), which was primarily driven by the response to 2 µM bleomycin. In contrast, no synergistic interaction was observed in Huh6 or Huh7 cells. Together, these findings indicate that the response to combined bleomycin/panobinostat treatment is strongly dependent on the molecular p53-family context. They therefore support a central role of p53 pathway integrity in the apoptosis induced by this combination.

### 3.3. Dual Targeting of Bcl-XL and Mcl-1 by Panobinostat Underlies Synergistic Apoptosis Induced by Bleomycin and Panobinostat

Targeting multiple anti-apoptotic proteins concurrently has been proposed as an effective strategy to overcome both intrinsic and acquired resistance to anticancer therapy [49]. Inhibition of Bcl-2 family members facilitates mitochondrial outer membrane permeabilization (MOMP) through cytochrome c release, representing a decisive initiating event of the intrinsic apoptotic cascade [50]. Among these proteins, myeloid cell leukemia-1 (Mcl-1) is a central determinant of tumor cell survival and therapy resistance and has therefore emerged as a prominent therapeutic target [51]. However, expression of Bcl-XL has been shown to modulate the efficacy of Mcl-1-directed therapies [52]. In addition, histone deacetylase inhibitors (HDACis) have been reported to dysregulate BH3 family proteins across diverse malignancies, thereby influencing apoptotic sensitivity [50].

We observed that both bleomycin and panobinostat induced apoptosis in HepG2 cells (Figure 1). To delineate the molecular basis of this effect, we examined key regulators of the intrinsic mitochondrial apoptotic pathway. Expression of the anti-apoptotic proteins Bcl-XL and Mcl-1 was assessed as indicators of mitochondrial involvement.

**Figure 2:**
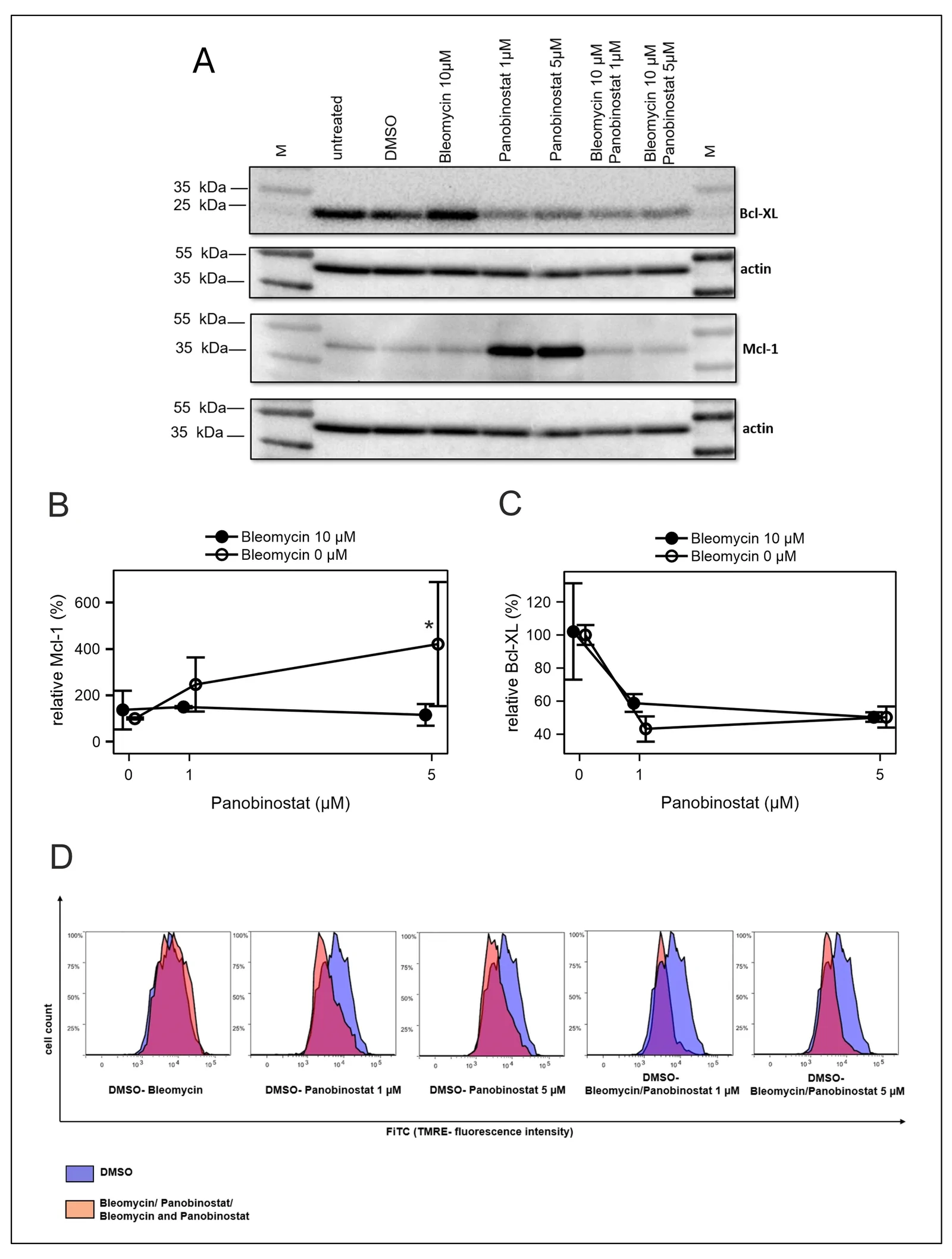
Bleomycin and panobinostat trigger apoptosis through the intrinsic, mitochondrial pathway in a synergistic manner. (**A**–**C**) HepG2 cells were incubated with bleomycin (10 µM), panobinostat (1 or 5 µM) or a combination of both for 24 h. DMSO was used as vehicle control. (**A**) Regulation of Bcl-XL and Mcl-1 was analyzed by Western blot. Shown are representative Western blots of three replicates; M = marker. (**B**,**C**) Densitometric analysis of Western blots showing relative amounts of Mcl-1 and Bcl-XL in HepG2 normalized to actin (*n* = 3, mean ± SD, Ordinary one-way ANOVA; Dunett’s multiple comparisons test; ns: *p* > 0.05, *: *p* = <0.05, **: *p* = <0.01 and ***: *p* = <0.001). (**D**) Analysis of mitochondrial outer membrane potential using TMRE-staining of HepG2 cells after 24 h of treatment with bleomycin or/and panobinostat. Representative TMRE fluorescence histograms from flow cytometry showing mitochondrial membrane potential differences between experimental groups (DMSO, blue; treatment, pink) at indicated concentrations, as single or combined treatments (*n* = 3).

In line with an anti-apoptotic response, panobinostat alone increased Mcl-1 levels in a dose-dependent manner, with a pronounced induction at 5 µM under control conditions (Bleo 0 µM; +321.5 percentage points vs. 0 µM panobinostat, *p* = 0.0086). In contrast, this panobinostat-driven Mcl-1 upregulation was effectively abolished in the presence of bleomycin (Bleo 10 µM), where Mcl-1 levels remained close to baseline despite panobinostat (Pano 5 µM vs. 0 µM at Bleo 10 µM: −20.7 percentage points, *p* = 0.843). Consistently, a pre-specified difference-in-differences analysis demonstrated a significant negative interaction at 5 µM panobinostat (DiD = −342.2 percentage points; 95% CI −658.0 to −26.4; *p* = 0.036), indicating that bleomycin counteracts the panobinostat-induced Mcl-1 increase beyond simple additivity. At 1 µM panobinostat, the interaction estimate was also negative (DiD = −133.8 percentage points) but did not reach statistical significance given the high variability (95% CI −449.6 to 182.1; *p* = 0.374) (Figure 2B).

Panobinostat markedly reduced Bcl-XL levels independent of bleomycin. In the factorial model, panobinostat showed a strong main effect on relative Bcl-XL (%) (*p* < 0.0001), whereas bleomycin alone had no significant effect (*p* = 0.352). Importantly, there was no evidence for non-additivity between bleomycin and panobinostat (bleomycin × panobinostat interaction: F(2,12) = 0.63, *p* = 0.5469), indicating that the panobinostat effect on Bcl-XL was comparable in the absence and presence of bleomycin. Consistently, panobinostat significantly decreased Bcl-XL at both concentrations under control conditions (Bleo 0 µM: 1 µM vs. 0 µM, −56.9 percentage points, *p* = 0.0002; 5 µM vs. 0 µM, −49.8 percentage points, *p* = 0.0006) and with bleomycin (Bleo 10 µM: 1 µM vs. 0 µM, −43.3 percentage points, *p* = 0.0016; 5 µM vs. 0 µM, −51.8 percentage points, *p* = 0.0004). Difference-in-differences contrasts confirmed the absence of dose-specific interaction effects (*p* = 0.3839 for 1 µM; *p* = 0.8978 for 5 µM) (Figure 2C).

To directly assess mitochondrial integrity, we measured changes in mitochondrial membrane potential using TMRE staining. TMRE selectively accumulates in polarized mitochondria, whereas MOMP results in depolarization and loss of fluorescence. Bleomycin alone did not significantly alter membrane potential, while panobinostat induced a moderate, dose-independent depolarization (Figure 2D). In contrast, combined treatment resulted in a pronounced loss of mitochondrial membrane potential, confirming a pronounced induction of MOMP (Figure 2D).

In summary, panobinostat downregulated the anti-apoptotic protein Bcl-XL, whereas bleomycin had no measurable impact on Bcl-XL abundance. Factorial modeling demonstrated a strong main effect of panobinostat (*p* < 0.0001) without evidence of bleomycin–panobinostat non-additivity (interaction *p* = 0.5469; difference-in-differences *p* > 0.38), indicating that panobinostat-mediated Bcl-XL suppression is bleomycin-independent and consistent across conditions. In parallel, panobinostat triggered a compensatory, dose-dependent increase in the anti-apoptotic factor Mcl-1 under control conditions; importantly, this potentially pro-survival adaptation was neutralized by bleomycin, with a significant negative interaction at 5 µM (DiD = −342.2 percentage points; 95% CI −658.0 to −26.4; *p* = 0.036). Thus, while panobinostat lowers Bcl-XL, bleomycin concurrently prevents Mcl-1 upregulation, a pattern consistent with limiting anti-apoptotic escape signaling and potentially reducing treatment resistance. Thus, their combined activity—characterized by downregulation of Bcl-XL and suppression of compensatory Mcl-1 induction—removes critical anti-apoptotic barriers, thereby amplifying mitochondrial dysfunction and promoting tumor cell death.

### 3.4. Panobinostat-Driven Caspase-9 Activation in HepG2 Cells

**Figure 3:**
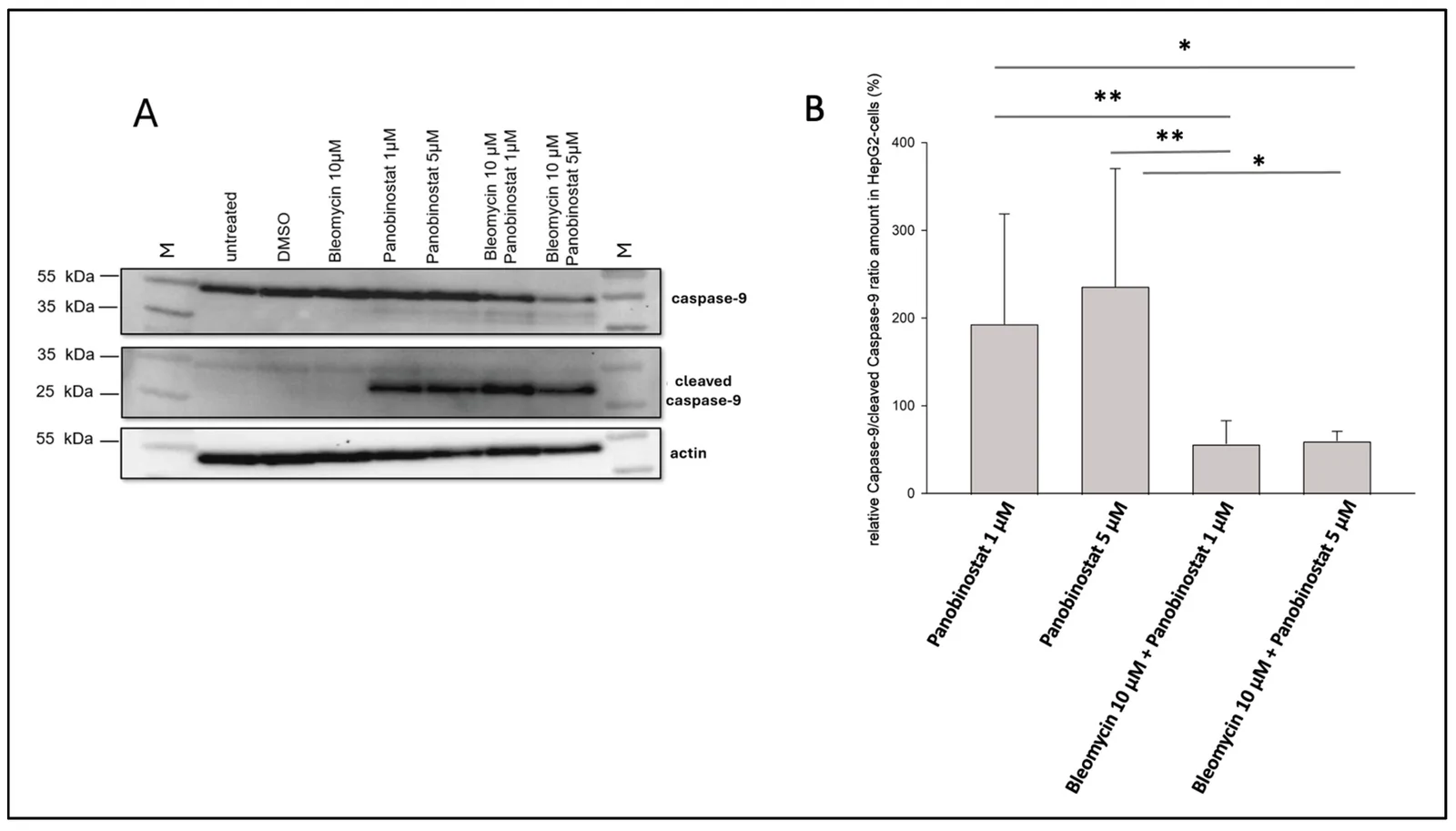
Combined treatment with bleomycin and panobinostat Induces Caspase-9 Cleavage. HepG2 cells were incubated with bleomycin (10 µM), panobinostat (1 or 5 µM) or a combination of both for 24 h. DMSO was used as vehicle control. Processing of caspase-9 (**A**) was analyzed by Western blot. Shown are representative Western blots of 3 replicates. Densitometric analysis (**B**) of Western blot shown is the relative amount of cleaved caspase-9 in HepG2 normalized to actin (*n* = 3) (mean ± SD, Welch’s *t*-test; *: *p* = <0.05, **: *p* = <0.01 and ***: *p* = <0.001). M = marker.

Destabilization of the mitochondrial membrane, as demonstrated in Figure 2, promotes the release of cytochrome c into the cytosol and subsequent activation of the initiator caspase-9. Although bleomycin has been reported to induce caspase-9 processing in other cellular models [53], this effect was not detectable in HepG2 cells under our experimental conditions (Figure 3). In contrast, panobinostat monotherapy induced caspase-9 cleavage, confirming activation of the intrinsic apoptotic pathway.

Combined treatment with bleomycin and panobinostat resulted in pronounced caspase-9 cleavage (Figure 3).

### 3.5. Combined Bleomycin and Panobinostat Treatment Enhances Caspase-8-Associated Apoptotic Signaling

**Figure 4:**
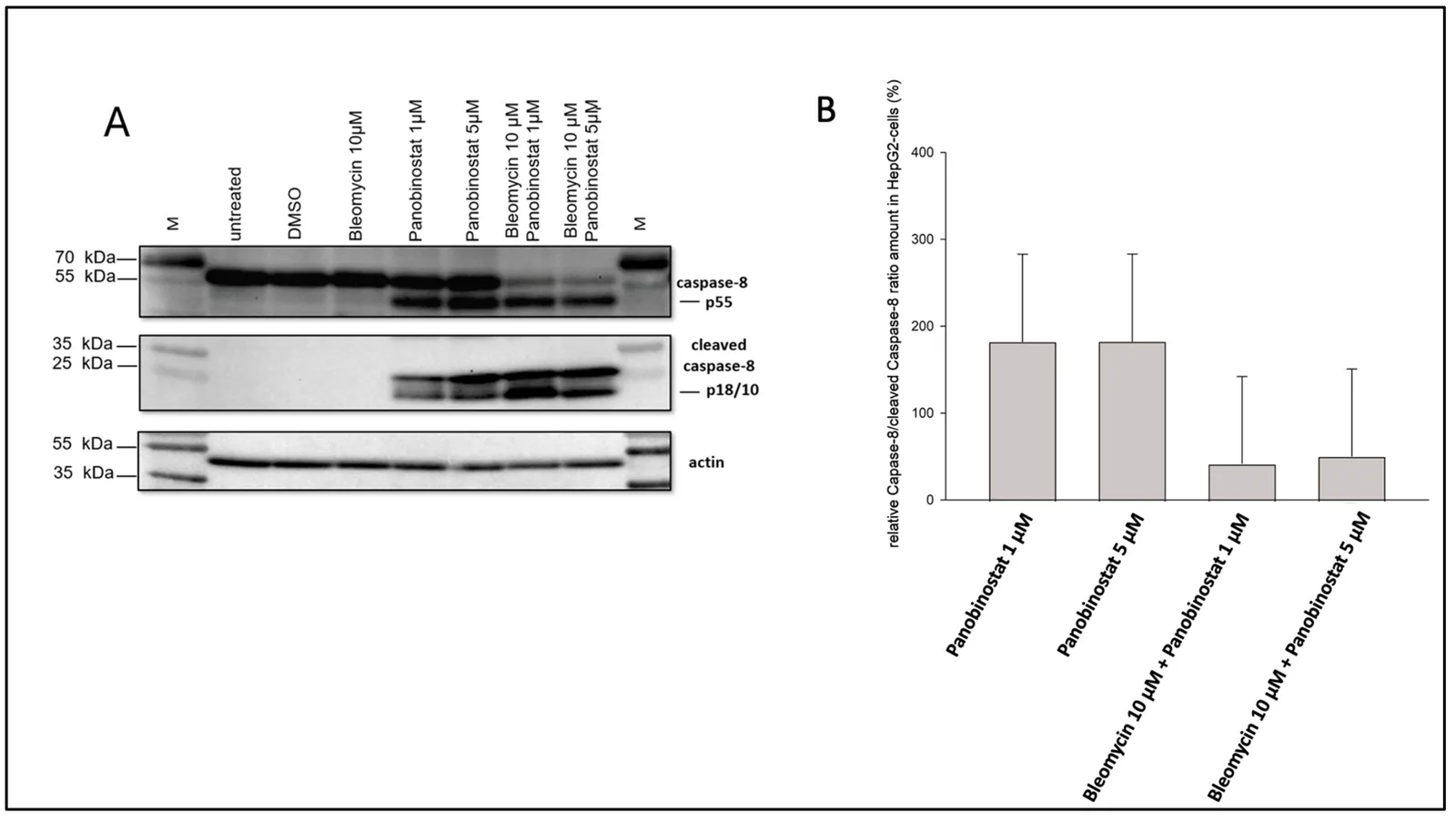
Combined treatment with bleomycin and panobinostat enhances apoptosis associated with caspase-8 activation. HepG2 cells were incubated with bleomycin (10 µM), panobinostat (1 or 5 µM) or a combination of both for 24 h. DMSO was used as vehicle control. Processing of caspase-8 (**A**) was analyzed by Western blot. Shown are representative Western blots of 3 replicates. Densitometric analysis (**B**) of Western blot shown is the relative amount of cleaved caspase-8 in HepG2 normalized to actin (*n* = 3). M = marker.

To further delineate the molecular mechanisms underlying the enhanced cytotoxicity of combined bleomycin and panobinostat treatment, we next investigated caspase-8 cleavage as a downstream indicator of caspase-8-associated apoptotic signaling. Caspase-8 is commonly activated in death receptor-associated apoptosis, although cleavage of caspase-8 alone does not formally prove canonical death receptor activation at the level of receptor clustering or DISC formation. Panobinostat monotherapy triggered caspase-8 activation, as evidenced by the appearance of the catalytically active p18 and p10 subunits. Of note, co-treatment with bleomycin further potentiated this effect, resulting in a marked accumulation of active caspase-8 fragments (Figure 4).

These findings demonstrate that panobinostat—and more prominently its combination with bleomycin—engages caspase-8-associated apoptotic signaling in HepG2 cells. Together with the observed activation of caspase-9, caspase-8 activation likely contributes to the amplified apoptotic response elicited by combined treatment. This is consistent with our previous work showing that bleomycin-induced p53 upregulation promotes transcriptional activation of the CD95 gene, thereby enhancing CD95 expression and sensitizing HCC cell lines to CD95-mediated apoptosis [25,26,54]. However, direct receptor-proximal events such as CD95/Fas expression, FADD recruitment, and DISC formation were not addressed in the present study.

### 3.6. Mcl-1 Modulates the Apoptotic Effect of Panobinostat and Combined Bleomycin and Panobinostat Treatment in HepG2 Cells

To examine whether the observed changes in Mcl-1 expression affected the apoptotic response, HepG2 cells were subjected to siRNA-mediated Mcl-1 knockdown or transient Mcl-1 overexpression.

**Figure 5:**
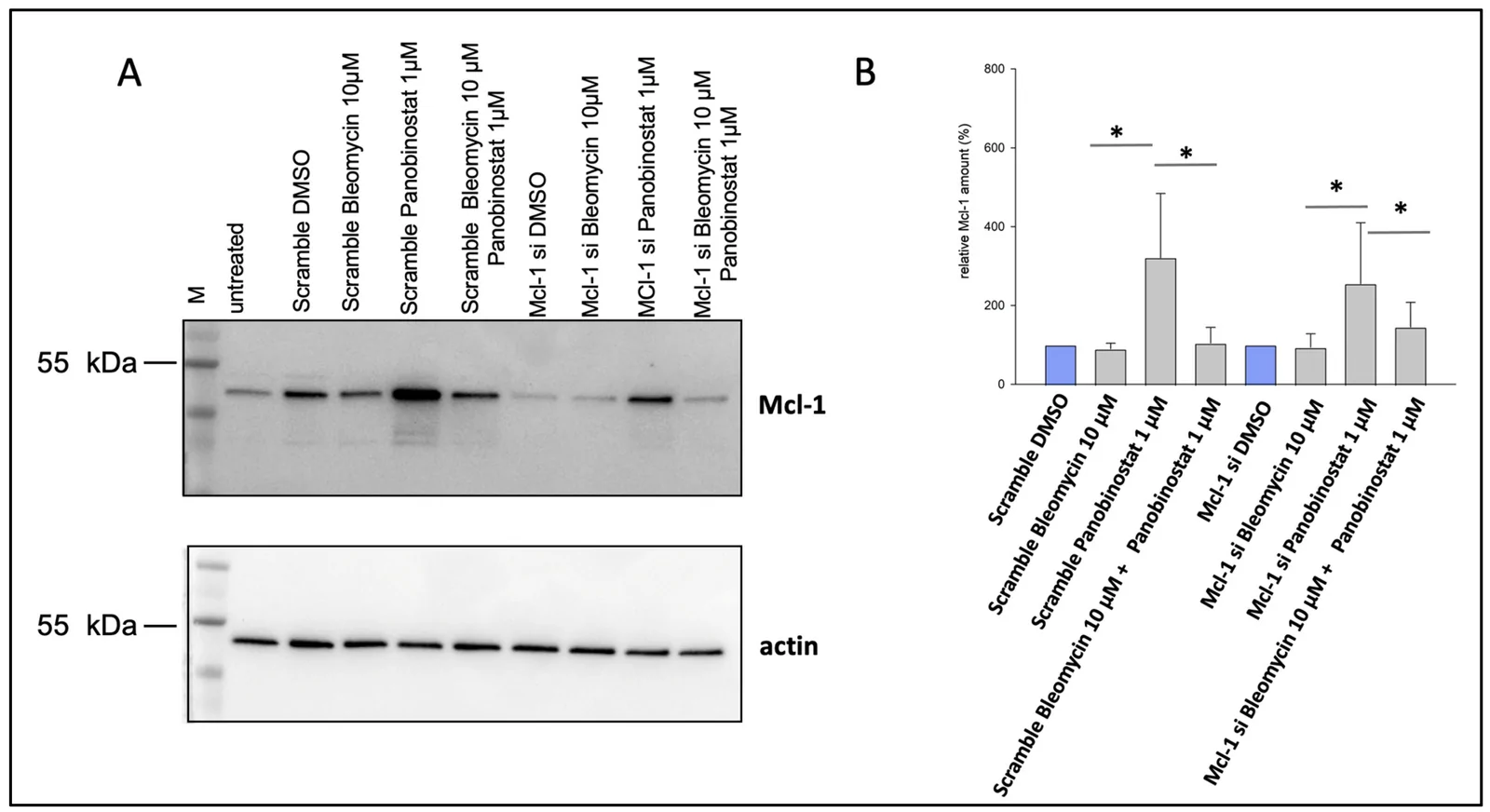
MCl-1 knockdown reduces Mcl-1 when combining panobinostat and bleomycin. HepG2 cells were incubated with bleomycin (10 µM), panobinostat (1 µM) or a combination of both for 24 h. DMSO was used as vehicle control. Processing of Mcl-1 (**A**) was analyzed by Western blot. Shown are representative Western blots of 3 replicates. Densitometric analysis (**B**) of Western blot shown is the relative amount of Mcl-1 in HepG2 normalized to actin (*n* = 3) (mean ± SD, Welch’s *t*-test; *: *p* = <0.05, **: *p* = <0.01 and ***: *p* = <0.001). M = marker. si = silencing.

**Figure 6:**
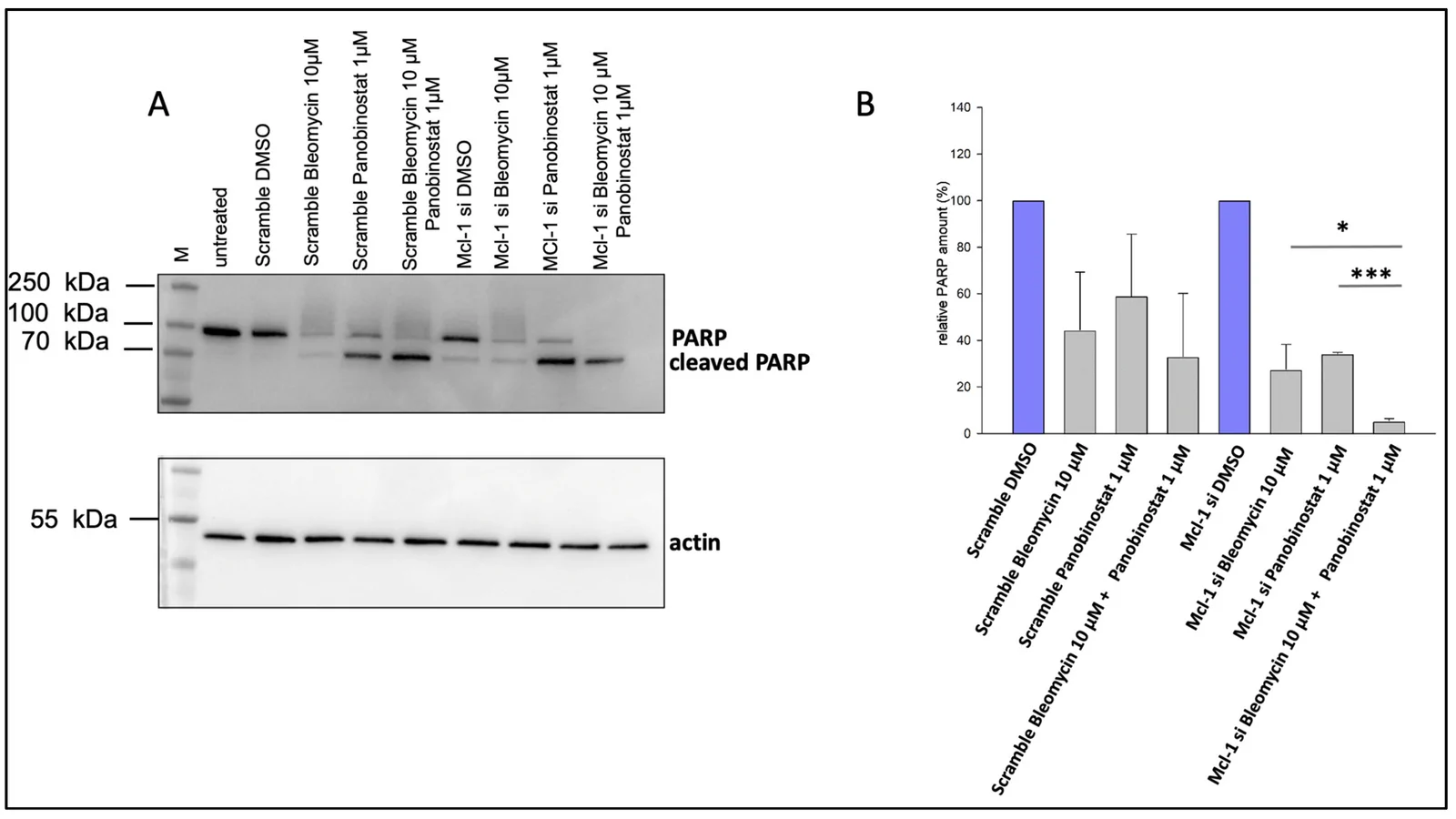
Mcl-1 knockdown is associated with increased PARP cleavage after panobinostat and the combination of panobinostat and bleomycin treatment. HepG2 cells were incubated with bleomycin (10 µM), panobinostat (1 µM) or a combination of both for 24 h. DMSO was used as vehicle control. Processing of PARP (**A**) was analyzed by Western blot. Shown are representative Western blots of 3 replicates. Densitometric analysis (**B**) of Western blot shown is the relative amount of Mcl-1 in HepG2 normalized to actin (*n* = 3) (mean ± SD, Welch’s *t*-test; *: *p* = <0.05, **: *p* = <0.01 and ***: *p* = <0.001). M = marker. EV = empty vector, oe = overexpression.

In cells transfected with scrambled control siRNA, panobinostat increased Mcl-1 protein levels, whereas this increase was not observed when panobinostat was combined with bleomycin, consistent with the findings shown in Figure 2. Transfection with Mcl-1-specific siRNA markedly reduced Mcl-1 protein levels under the respective treatment conditions (Figure 5). Compared with the corresponding scrambled siRNA controls, Mcl-1 knockdown was associated with increased PARP cleavage after panobinostat treatment and after combined panobinostat and bleomycin treatment (Figure 6). These results indicate that reduced Mcl-1 expression increases the apoptotic response to both treatments.

**Figure 7:**
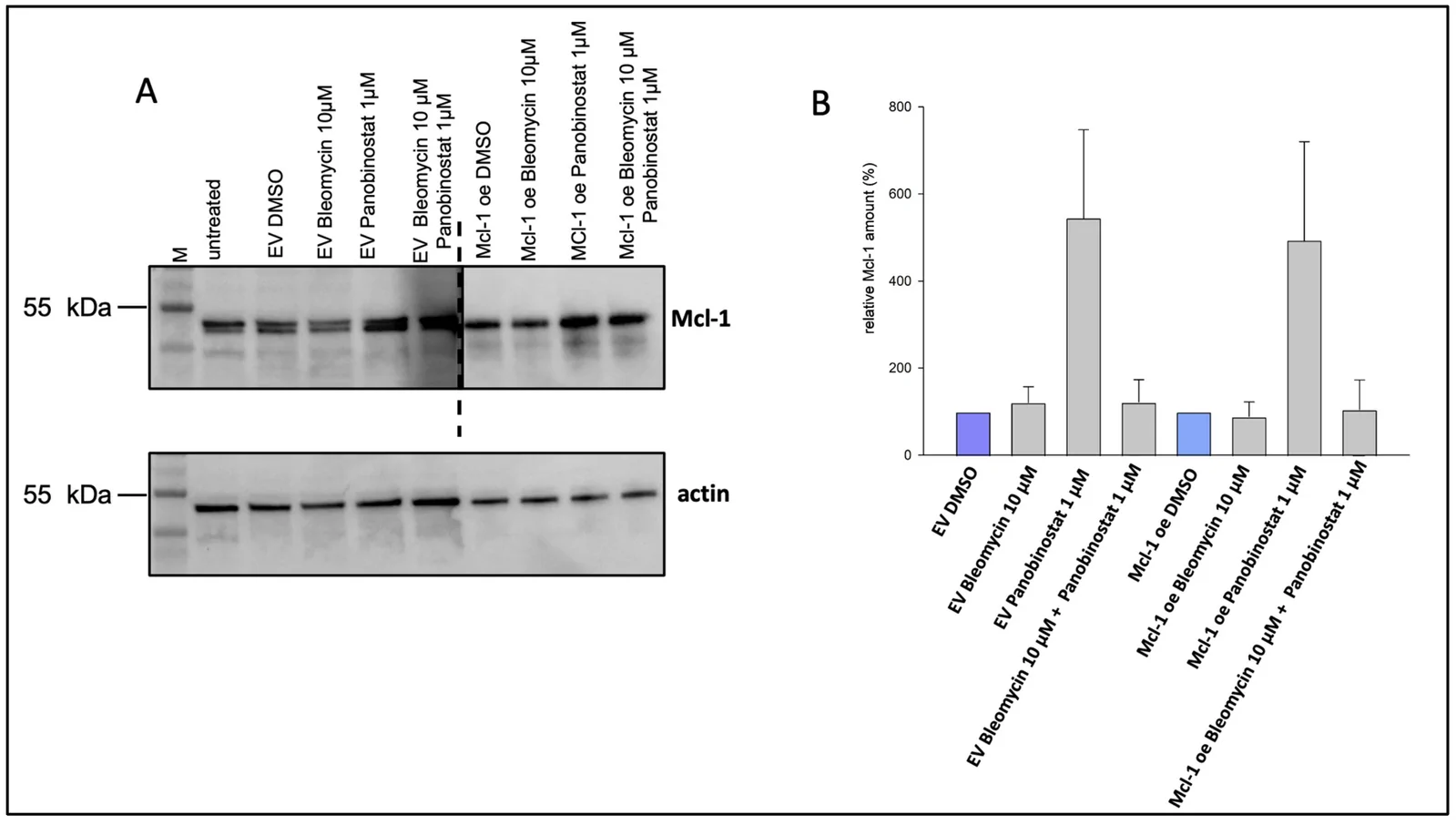
MCl-1 overexpression increases Mcl-1 when combining panobinostat and bleomycin. HepG2 cells were incubated with bleomycin (10 µM), panobinostat (1 µM) or a combination of both for 24 h. DMSO was used as vehicle control. Processing of Mcl-1 (**A**) was analyzed by Western blot. Shown are representative Western blots of 3 replicates. Densitometric analysis (**B**) of Western blot shown is the relative amount of Mcl-1 in HepG2 normalized to actin (*n* = 3) (mean ± SD). M = marker. EV = empty vector, oe = overexpression.

**Figure 8:**
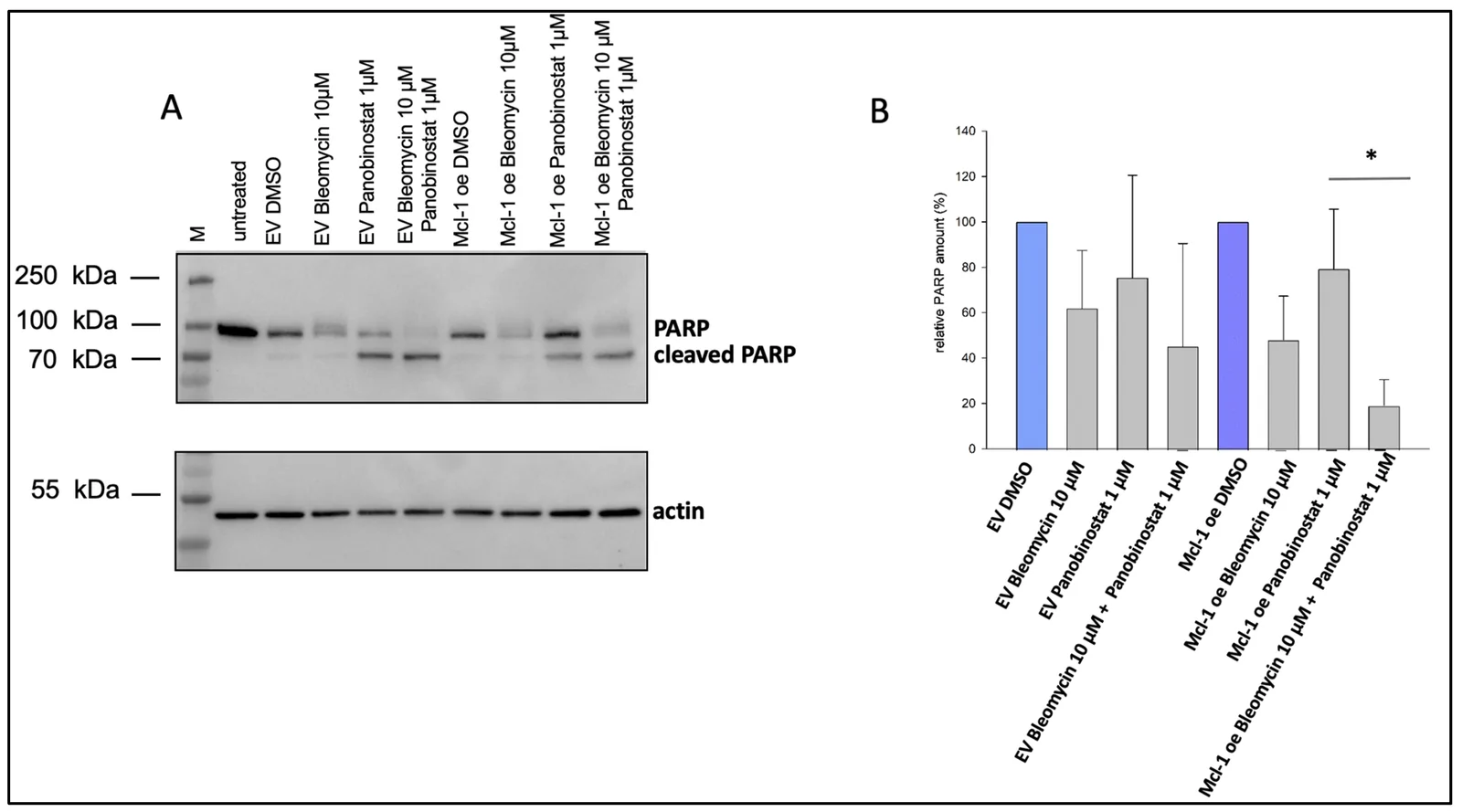
Mcl-1 overexpression reduces PARP cleavage after panobinostat treatment and attenuates PARP cleavage under combined bleomycin and panobinostat treatment. HepG2 cells were incubated with bleomycin (10 µM), panobinostat (1 µM) or a combination of both for 24 h. DMSO was used as vehicle control. Processing of PARP (**A**) was analyzed by Western blot. Shown are representative Western blots of 3 replicates. Densitometric analysis (**B**) of Western blot shown is the relative amount of Mcl-1 in HepG2 normalized to actin (*n* = 3) (mean ± SD, Welch’s *t*-test; *: *p* = <0.05, **: *p* = <0.01 and ***: *p* = <0.001). M = marker. EV = empty vector, oe = overexpression.

In a complementary approach, transient transfection with an Mcl-1 expression vector increased Mcl-1 protein levels compared with the corresponding empty vector control (Figure 7). Mcl-1 overexpression reduced PARP cleavage after panobinostat treatment and attenuated PARP cleavage following combined panobinostat and bleomycin treatment (Figure 8). Thus, increased Mcl-1 expression partially protected HepG2 cells from treatment-induced apoptotic processing.

### 3.7. A Caspase-3-Dependent Execution Phase Underlies Combined Treatment-Driven Apoptotic Synergy

**Figure 9:**
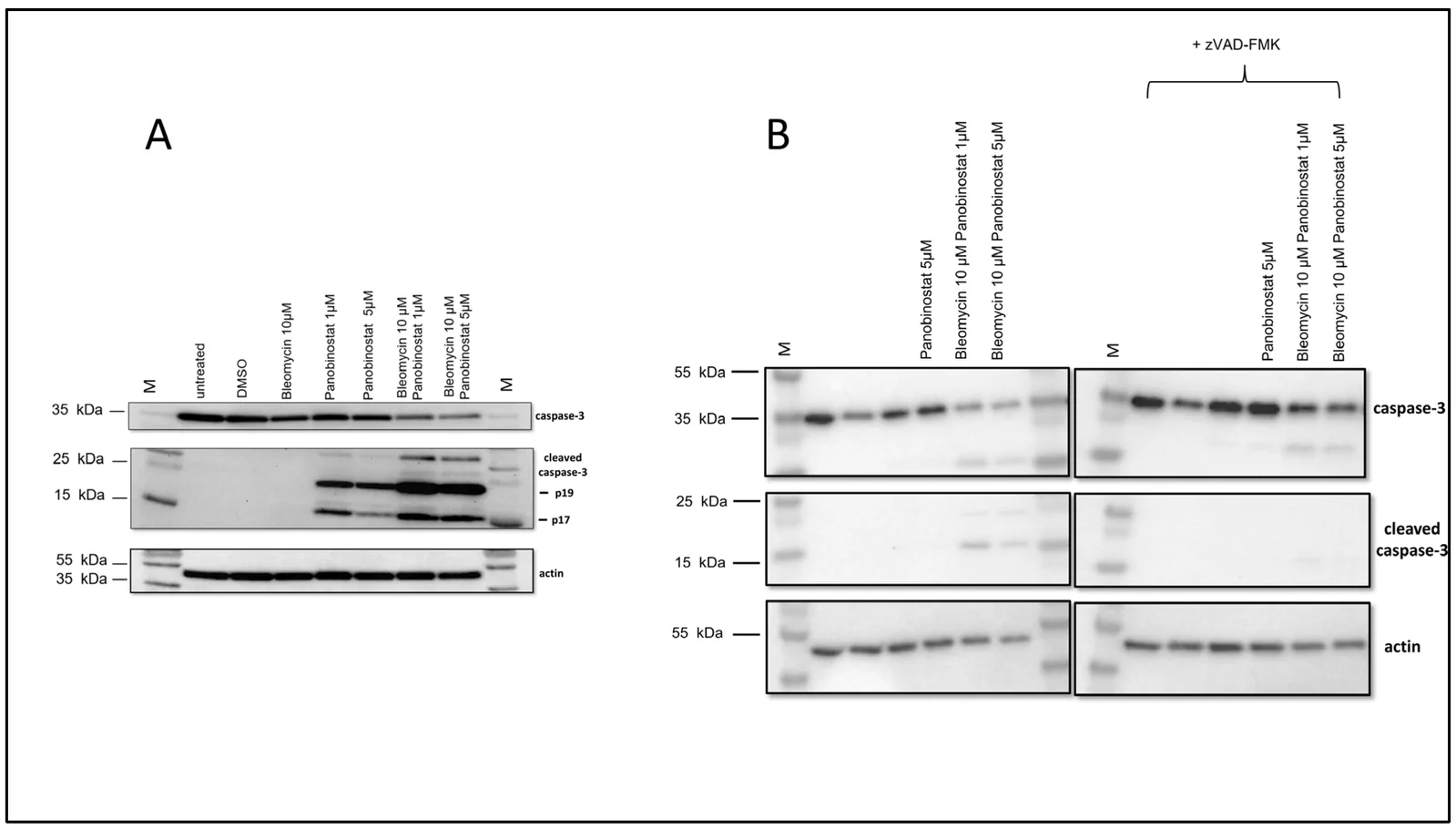

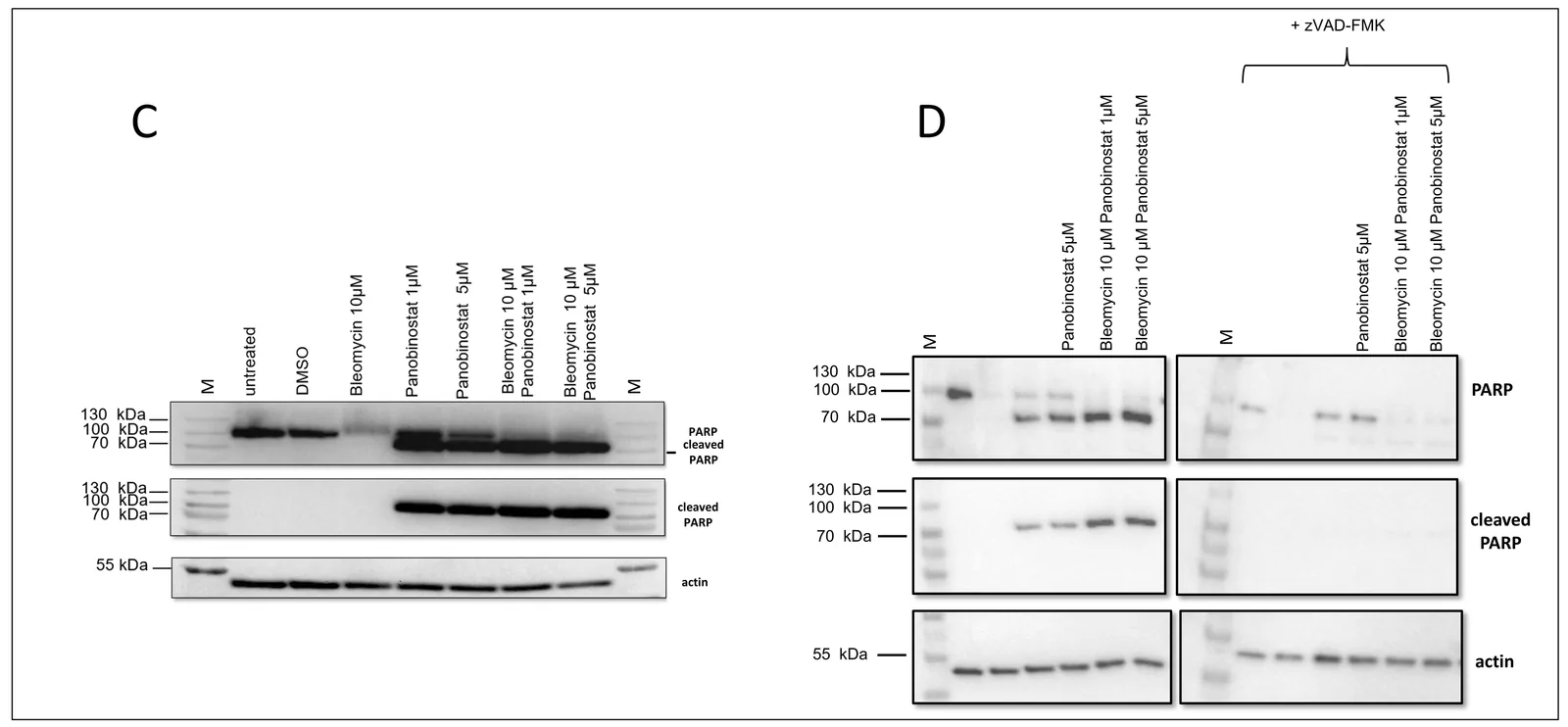
Caspase-3 and PARP Processing under Treatment with bleomycin and/or panobinostat and its Inhibition by zVAD-FMK. (**A**,**C**) HepG2 cells were incubated with bleomycin (10 µM), panobinostat (1 or 5 µM) or a combination of both for 24 h. DMSO was used as vehicle control. Processing of (**A**) caspase-3 and (**C**) PARP was analyzed by Western blot. Shown are representative Western blots of 3 replicates. (**B**,**D**) Activation of caspase-3 and PARP can effectively be blocked by zVAD-FMK. HepG2 cells were treated with either one bleomycin or panobinostat alone or a combination of both, as well as zVAD-FMK. DMSO was used as vehicle control. Processing of (**B**) caspase-3 and (**D**) PARP was analyzed by Western blot. Shown are representative Western blots of 3 replicates. M = marker.

Effector caspase-3 represents the central executioner of apoptosis and is commonly regarded as the “point of no return” within the apoptotic cascade [55]. To determine whether the enhanced cytotoxicity observed upon combined treatment correlates with activation of the execution phase of apoptosis, we analyzed proteolytic processing of pro-caspase-3 into its active subunits. Bleomycin monotherapy did not induce detectable caspase-3 cleavage, whereas panobinostat alone resulted in moderate caspase-3 activation. In contrast, co-treatment with bleomycin and panobinostat led to pronounced caspase-3 cleavage, indicating robust activation of the effector caspase (Figure 9A).

Poly(ADP-ribose) polymerase (PARP), a nuclear enzyme involved in DNA repair, is a well-established downstream substrate of caspase-3 during apoptosis. Consistent with the caspase-3 activation profile, PARP processing was not observed following bleomycin monotherapy. Panobinostat treatment induced substantial PARP cleavage, which was further enhanced in the presence of bleomycin (Figure 9C).

To confirm that the amplified apoptotic response elicited by combined treatment is caspase-dependent, HepG2 cells were pre-treated with the pan-caspase inhibitor zVAD-FMK prior to exposure to bleomycin, panobinostat, or their combination. zVAD-FMK markedly reduced levels of cleaved caspase-3 and cleaved PARP in panobinostat-treated cells and similarly suppressed cleavage under combined treatment conditions, confirming effective inhibition of caspase-dependent apoptosis (Figure 9B,D).

Collectively, these findings demonstrate that the synergistic apoptotic response induced by the bleomycin–panobinostat combination is mediated predominantly through caspase-dependent pathways.

## 4. Discussion

In the context of current guideline-recommended systemic therapies, which are dominated by immune checkpoint inhibitor-based combinations and tyrosine kinase inhibitors, durable tumor control in HCC remains limited by intrinsic and acquired resistance mechanisms [11,56,57,58].

Our data identify apoptosis resistance at the mitochondrial checkpoint as a critical mechanism that can be therapeutically targeted through rational epigenetic priming. We demonstrate that panobinostat lowers mitochondrial anti-apoptotic barriers via Bcl-XL suppression but simultaneously induces a compensatory Mcl-1 survival response, which is effectively neutralized by bleomycin (Figure 6). This dual modulation, together with enhanced extrinsic apoptotic signaling, converges on robust caspase-3-dependent execution and explains the synergistic induction of apoptosis. Our findings uncover a previously unrecognized mechanistic interaction between a pan-HDAC inhibitor and a DNA-damaging agent in HCC cells and provide a strong rationale for developing apoptosis-targeted, epigenetically guided combination strategies. Importantly, the conclusions of the present study should be interpreted within the boundaries of the experimental system used. The mechanistic data were primarily generated in HepG2 cells, which represent a p53-functional HCC cell model. Therefore, our findings provide a mechanistic proof of concept that the bleomycin–panobinostat combination can lower the apoptotic threshold in this defined cellular context, rather than a broadly generalizable conclusion for apoptosis resistance in HCC as a whole. HCC is molecularly heterogeneous, and apoptotic sensitivity may differ substantially according to p53 status, BCL-2 family protein expression, differentiation state, and etiological background. Accordingly, the broader applicability of this combination strategy requires validation in additional HCC models, including p53-mutant and p53-deficient cell lines, patient-derived organoids, and in vivo systems.

The application of bleomycin is well established in locoregional therapy of HCC via TACE. We have previously shown that bleomycin induces extrinsic and intrinsic apopto-sis signaling pathways in HCC cell lines [24,25,56,58,59].

In the present study, caspase-8 cleavage was used as a downstream readout of caspase-8-associated/extrinsic apoptosis-related signaling. We acknowledge that receptor-proximal events were not directly analyzed and that our data therefore do not formally demonstrate canonical CD95/Fas–DISC pathway activation. Definitive proof of death receptor pathway activation would require additional experiments, including assessment of CD95/Fas surface expression, receptor clustering, FADD recruitment, and DISC formation. However, the primary mechanistic focus of this study was the cooperative regulation of apoptotic execution and mitochondrial checkpoint control by bleomycin and panobinostat. Within this framework, the combined detection of caspase-8 processing, caspase-9 activation, caspase-3/PARP cleavage, mitochondrial depolarization, modulation of Bcl-XL and Mcl-1, and zVAD-FMK-sensitive cell death supports the conclusion that caspase-8-associated signaling contributes to the amplified apoptotic response. Future studies should define whether CD95/Fas–DISC formation is directly involved in this process.

Here we demonstrate synergistic induction of apoptosis in HepG2 cells when bleomycin (2–10 µM; 0.0028–0.014 mg/mL) was combined with the HDAC inhibitor panobinostat (1–5 µM; 350–1750 ng/mL). Within this defined p53-functional model system, this interaction supports a dose-de-escalation concept: rather than relying on higher single-agent exposures, synergistic co-treatment may shift the effective response into a lower-dose window while amplifying apoptotic tumor cell killing. At this stage, however, this concept should be considered hypothesis-generating and requires confirmation across additional HCC models and pharmacologically relevant experimental systems.

Conceptually, synergy provides leverage to “trade” concentration for mechanism—engaging convergent pro-apoptotic pathways more efficiently than either agent alone—thereby permitting substantial lowering of both compounds to reach a comparable biological endpoint.

However, bleomycin-associated pulmonary toxicity remains a major translational limitation and must be considered a primary safety concern for any bleomycin-based treatment concept. Bleomycin can cause potentially severe lung injury, including pneumonitis and pulmonary fibrosis, and this risk is influenced by cumulative dose, age, pre-existing lung disease, renal function, oxygen exposure, and previous or concomitant therapies. Therefore, any future clinical development of a bleomycin–panobinostat combination would require stringent patient selection, baseline pulmonary assessment, careful monitoring for early respiratory symptoms, and follow-up evaluation of pulmonary function and radiological changes. Preventive strategies would need to focus on minimizing cumulative bleomycin exposure, avoiding unnecessary oxygen exposure when possible, and considering liver-directed delivery approaches that limit systemic exposure. Importantly, the present in vitro study cannot assess organ-specific toxicities such as bleomycin-induced lung injury. Future preclinical studies must therefore determine whether the observed apoptotic synergy can be maintained at substantially reduced bleomycin doses or through locoregional delivery, and whether bleomycin-sparing or bleomycin-mimetic strategies may preserve the relevant DNA damage/apoptosis circuitry while reducing pulmonary risk.

In this context, efficacy might be maintained at reduced exposures through combination therapy, and the therapeutic index may be improved, especially when coupled to liver-directed delivery approaches or bleomycin-mimetic agents intended to preserve the relevant DNA damage/apoptosis circuitry with less systemic burden. A major limitation of bleomycin is the drug-induced lung injury (DILI), particularly interstitial pulmonary fibrosis. This undesirable effect encounters in 10% of cases. The incidence of DILI depends on a variety of risk factors, like cumulative drug dose, underlying malignant disease, or concurrent radiation. Bleomycin causes cell toxicity, and the mechanisms are well described based on in vitro experiments on DNA. Interstitial edema with an influx of inflammatory and immune cells has been described in DILI after bleomycin administration, which may lead to pulmonary fibrosis. Clinical symptoms are unspecific for bleomycin-induced lung injury. Patients receiving bleomycin who encounter dyspnea or dry cough should be suspected of developing DILI and therefore should be closely monitored. Computed tomography can help in the diagnosis of DILI in this context.

Most efficiently, bleomycin-induced DILI could be averted by avoiding cumulative toxicity. It is recommended to stop bleomycin if DILI is suspected.

All patients receiving bleomycin must be monitored regularly from the clinical and radiological point of view. Treatment of bleomycin-induced DILI consists of steroids, and depending on the severity other drugs like antibiotics, beta agonists, and anticholinergics, or inotropic agents could improve the clinical condition.

Translationally, these data motivate follow-up studies that (i) quantify the extent of dose reduction compatible with maintained synergy across additional HCC models and (ii) integrate pharmacokinetic constraints to identify clinically achievable combination exposures.

**Figure 10:**
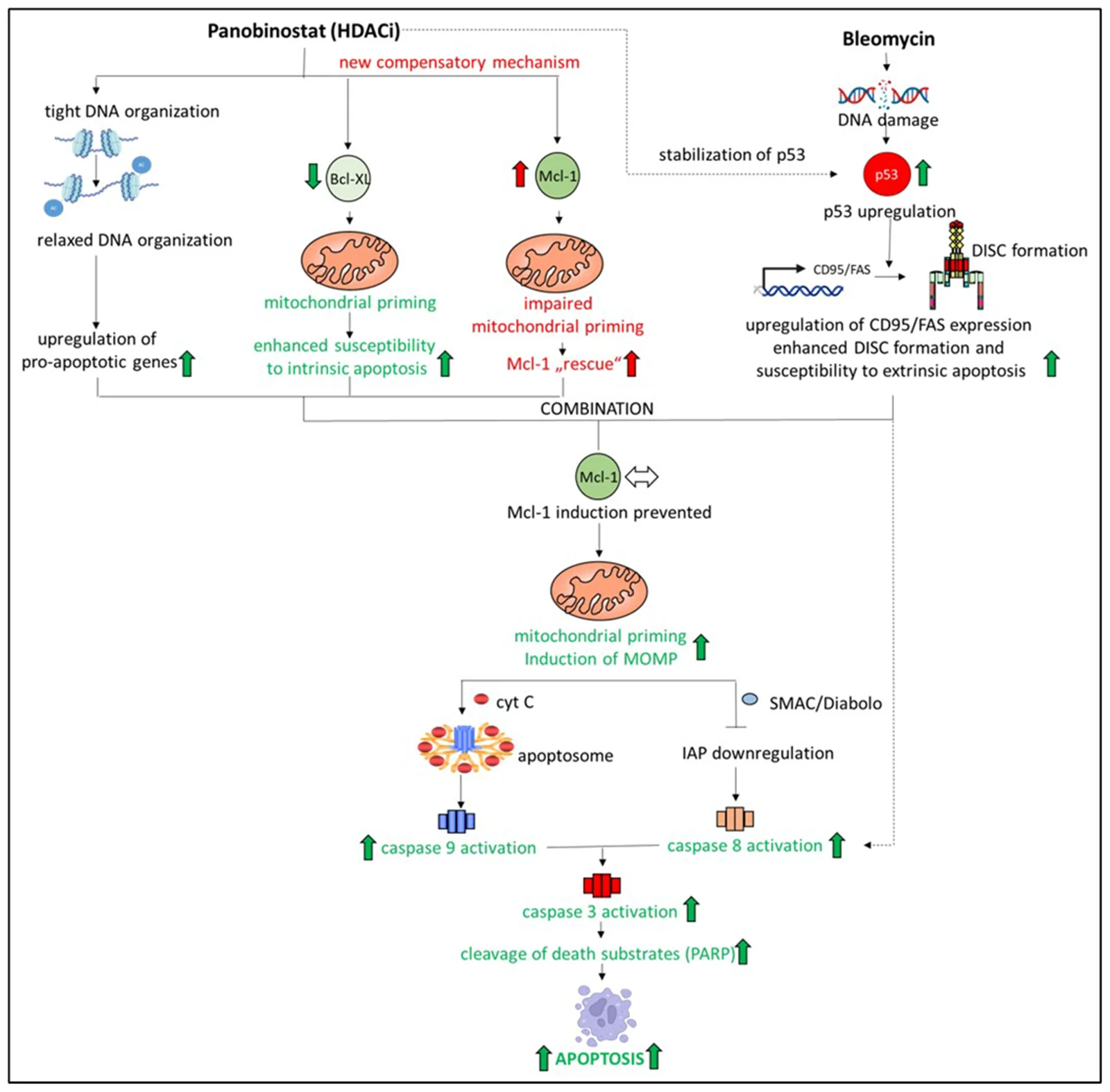
Mechanistic model of bleomycin/panobinostat-induced apoptotic synergy in HepG2 cells. The figure summarizes the HepG2-based working model derived from the present study and integrates se-lected literature-supported mechanisms. Panobinostat induces chromatin relaxation and suppresses the anti-apoptotic protein BCL-XL, thereby inducing pronounced mitochondrial priming while concomitantly eliciting a compensatory upregulation of MCL-1. Bleomycin induces DNA dam-age-driven p53 activation, which has been reported to result in transcriptional upregulation of the death receptor CD95 (Fas) and potentiation of caspase-8-associated apoptotic signaling [26,54,60]. In combination, HDAC inhibition by panobinostat may contribute to p53 stabilization [59,61], hereby potentially supporting bleomycin-induced p53 signaling and downstream caspa-se-8-associated apoptotic signaling. Importantly, bleomycin abrogates panobinostat-induced MCL-1 upregulation, effectively removing a key mitochondrial survival checkpoint in this model system. The coordinated engagement of intrinsic mitochondrial signaling and caspase-8-associated apoptotic signaling results in robust mitochondrial priming, mitochondrial outer membrane per-meabilization (MOMP), cytochrome c release, apoptosome assembly, and caspase-9 activation. In parallel, enhanced caspase-8 cleavage further amplifies caspase-3-dependent apoptotic execution. Both caspase-9 and caspase-8 converge on caspase-3-dependent execution of apoptosis. Pro-apoptotic pathways supported by the present data are shown in green, while the rescue mechanism is indicated in red. Green arrows = enhanced apoptosis; red arrows = reduced apoptosis. This schematic should be interpreted as a mechanistic working model and requires further valida-tion in additional HCC model systems. Green arrows 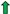 = enhanced apoptosis; red arrows 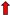 = reduced apoptosis).

In this study, we provide mechanistic evidence that bleomycin and panobinostat synergistically induce apoptosis in HepG2 HCC cells (Figure 10). While each agent alone triggered apoptotic features, the combination consistently amplified cell death and shifted cells toward a fully engaged execution phase. Mechanistically, the combined treatment lowered the anti-apoptotic barrier at the mitochondria by (i) downregulating Bcl-XL (driven by panobinostat and maintained in the combination) (Figure 2C) and (ii) preventing the compensatory Mcl-1 upregulation that occurred with panobinostat monotherapy (Figure 2B). This translated into a significant mitochondrial outer membrane permeabilization, evidenced by a pronounced loss of mitochondrial membrane potential (Figure 2D). Importantly, combined treatment potentiated caspase-8 activation, implicating extrinsic pathway engagement as an additional amplifier (Figure 4). Ultimately, the combination produced strong caspase-3 activation and PARP cleavage, and these effects were suppressed by zVAD-FMK, demonstrating that the synergistic induction of apoptosis is mediated predominantly through caspase-dependent apoptotic execution (Figure 9).

**The new findings are:**

1. Mechanistic synergy at the anti-apoptotic checkpoint:

We show that panobinostat triggers a compensatory Mcl-1 upregulation (a plausible resistance response), and that bleomycin prevents this adaptation, shifting the balance toward apoptosis. This “block the escape response” mechanism is a novel observation.

2. Extrinsic pathway engagement as an amplifier of a mitochondria-primed state:

The observation that combined treatment potentiates caspase-8 activation provides a second, reinforcing axis that plausibly helps drive the system to caspase-3/PARP execution.

3. Caspase dependence is experimentally demonstrated:

The zVAD-FMK experiments strengthen causal interpretation by showing that the enhanced phenotype is predominantly caspase-dependent, supporting a synergistic activation of the intrinsic and extrinsic apoptosis signaling pathways.

Our data demonstrate that bleomycin alone induces only limited cytotoxicity in HepG2 cells, whereas its combination with panobinostat produces a markedly synergistic apoptotic response. Mechanistically, this synergy is driven by coordinated disruption of mitochondrial anti-apoptotic barriers and activation of complementary apoptotic pathways. A central determinant of this effect is the regulation of Mcl-1, a key survival factor frequently overexpressed in HCC and strongly associated with therapeutic resistance. While panobinostat downregulates Bcl-XL and primes mitochondria for apoptosis, it simultaneously induces compensatory Mcl-1 upregulation, representing an adaptive resistance mechanism. Bleomycin effectively suppresses this Mcl-1 induction, thereby lowering the apoptotic threshold and enabling robust mitochondrial outer membrane permeabilization and caspase-dependent execution.

In addition to mitochondrial priming, combined treatment activates the extrinsic apoptotic pathway, as evidenced by enhanced caspase-8 cleavage. Convergence of intrinsic (caspase-9) and extrinsic (caspase-8) signaling results in pronounced caspase-3 activation and PARP cleavage, marking irreversible commitment to apoptosis. These findings provide a mechanistic explanation for the synergistic interaction between bleomycin and panobinostat and underscore the therapeutic potential of rational combinations that integrate DNA damage with epigenetic modulation to overcome apoptosis resistance in HCC.

HDAC inhibitors have emerged as promising epigenetic modulators capable of restoring apoptotic sensitivity in HCC. Preclinical studies indicate that HDAC inhibition disrupts oncogenic transcriptional programs and sensitizes tumor cells to cytotoxic stress. Our data extend these observations by demonstrating that panobinostat alone induces only moderate apoptosis but that its combination with bleomycin markedly amplifies apoptotic execution through dual caspase activation and suppression of Mcl-1-mediated survival signaling. Importantly, bleomycin neutralizes the panobinostat-induced Mcl-1 survival response, highlighting a complementary, non-redundant interaction between both agents.

Panobinostat is clinically approved for relapsed and refractory multiple myeloma and has demonstrated significant clinical benefit in combination regimens. In solid tumors, including HCC, resistance is frequently mediated by persistent expression of anti-apoptotic proteins such as Mcl-1 and Bcl-XL. Simultaneous targeting of these proteins represents an attractive therapeutic strategy. Our findings pharmacologically validate this concept by demonstrating that panobinostat and bleomycin cooperatively suppress both Mcl-1 and Bcl-XL, activate initiator and executioner caspases, and synergistically induce apoptosis in HCC cells.

Given the prior clinical application of bleomycin in TACE and emerging evidence supporting its efficacy in intermediate-stage HCC, our data provide a mechanistic rationale for further evaluation of bleomycin–HDAC inhibitor combinations in locoregional or liver-directed treatment strategies. Molecular profiling of Mcl-1 and Bcl-XL expressions may further enable patient stratification and precision-based therapeutic approaches.

The present study has several limitations that should be considered when interpreting the findings. First, the mechanistic experiments were primarily performed in HepG2 cells, which represent a p53-functional HCC cell model. Therefore, the results should be regarded as a mechanistic proof of concept in this defined experimental system rather than as a broadly generalizable conclusion for apoptosis resistance in HCC. Second, HCC is molecularly heterogeneous, and apoptotic sensitivity may differ depending on p53 status, BCL-2 family protein expression, differentiation state, etiological background, and additional resistance pathways. Third, the in vitro design does not reproduce the complexity of the tumor microenvironment, liver-specific drug exposure, pharmacokinetics, immune-cell interactions, or stromal influences. In addition, organ-specific toxicities, particularly bleomycin-associated pulmonary injury, cannot be evaluated in this experimental setting. This is clinically relevant because bleomycin-induced lung toxicity remains a major safety concern and requires careful patient selection, baseline and follow-up pulmonary assessment, monitoring for early respiratory symptoms, and strategies to minimize cumulative systemic exposure. Future studies should therefore validate the bleomycin–panobinostat combination in additional molecularly diverse HCC models, including p53-mutant and p53-deficient cell lines, patient-derived organoids, and in vivo systems, and should assess whether reduced dosing, liver-directed delivery, or bleomycin-sparing approaches can preserve apoptotic synergy while limiting systemic toxicity.

In conclusion, our data identify a cooperative apoptotic vulnerability to combined bleomycin and panobinostat treatment in HepG2 cells, a p53-functional HCC cell model. The combination suppresses compensatory Mcl-1 induction, reduces Bcl-XL expression, promotes mitochondrial dysfunction, and enhances caspase-dependent apoptotic execution. These findings support a mechanistic proof of concept in this defined experimental system and provide a rationale for further validation in molecularly diverse HCC models before broader preclinical or translational conclusions are drawn.

## Data Availability

The original contributions presented in this study are included in the article. Further inquiries can be directed to the corresponding author.
